# The Possible Neuroprotective Effect of Silymarin against Aluminum Chloride-Prompted Alzheimer’s-Like Disease in Rats

**DOI:** 10.3390/brainsci10090628

**Published:** 2020-09-11

**Authors:** Hanaa R. Aboelwafa, Attalla F. El-kott, Eman M. Abd-Ella, Hany N. Yousef

**Affiliations:** 1 Department of Biological and Geological Sciences, Faculty of Education, Ain Shams University, Cairo 11566, Egypt; hany_barsoum@edu.asu.edu.eg; 2 Biology Department, Faculty of Science, King Khalid University, Abha 61421, Saudi Arabia; elkottaf@yahoo.com; 3 Zoology Department, College of Science, Damanhour University, Damanhour 22511, Egypt; 4 Zoology Department, College of Science, Fayoum University, Fayoum 63514, Egypt; eman_abdella@yahoo.co.uk; 5 Biology Department, College of Science and Art, Al-Baha University, Al-Mandaq 65581, Saudi Arabia

**Keywords:** Alzheimer’s disease, silymarin, hippocampus, aluminum chloride, oxidative stress, histology, ultrastructure

## Abstract

Alzheimer’s disease (AD) is a worldwide rapidly growing neurodegenerative disease. Here, we elucidated the neuroprotective effects of silymarin (SM) on the hippocampal tissues of aluminum chloride (AlCl_3_)-induced Alzheimer-like disease in rats using biochemical, histological, and ultrastructural approaches. Forty rats were divided into control, SM, AlCl_3_, and AlCl_3_ + SM groups. Biochemically, AlCl_3_ administration resulted in marked elevation in levels of lipid peroxidation (LPO) and nitric oxide (NO) and decrease in levels of reduced glutathione (GSH), catalase (CAT), and superoxide dismutase (SOD). Moreover, AlCl_3_ significantly increased tumor necrosis factor-α (TNF-α), interleukin-1beta (IL-1β), and acetylcholinesterase (AChE) activities. Furthermore, myriad histological and ultrastructural alterations were recorded in the hippocampal tissues of AlCl_3_-treated rats represented as marked degenerative changes of pyramidal neurons, astrocytes, and oligodendrocytes. Additionally, some myelinated nerve fibers exhibited irregular arrangement of their myelin coats, while the others revealed focal degranulation of their myelin sheaths. Severe defects in the blood–brain barrier (BBB) were also recorded. However, co-administration of SM with AlCl_3_ reversed most of the biochemical, histological, and ultrastructural changes triggered by AlCl_3_ in rats. The results of the current study indicate that SM can potentially mend most of the previously evoked neuronal damage in the hippocampal tissues of AlCl_3_-kindled rats.

## 1. Introduction

Alzheimer’s disease (AD) is a worldwide rapidly developing disease, about 66 million people are expected to suffer from it by 2030 and by 2050 this number is predicted to increase up to 115 million [1]. AD is a multifactorial, age-linked advanced neurodegenerative disease that primarily influences adults from mid to late age. Episodic memory impairment is a prominent sign from the early stages of AD, besides progressive cognitive impairment, as well as functional and behavioral alterations, which have a major influence on the individuals’ ability to execute normal daily living activities [2]. Even though many executive functions such as attention, language, orientation, and judgment are affected, the most common manifestation of AD is a gradual loss of memory [3]. Pathologically, AD is commonly distinguished by tau protein aggregation, extracellular amyloid-β protein (Aβ) deposition, and intracellular precipitation of neurofibrillary tangles (NFTs), in addition to neuronal synapse and pyramidal neuron loss [4]. The hippocampus, a part of the brain essential for some aspects of learning and memory, is regarded as one of the most susceptible areas in the development of early AD and other neurodegenerative disorders [5].

Environmental heavy metals are well-recognized substances influencing brain development. Many investigations have demonstrated that heavy metals are correlated with neurodegenerative disorders such as AD and Parkinson’s disease (PD) [6]. Aluminum (Al) is one of the heavy metals involved in neurodegenerative disease development, as it affects several cellular metabolic pathways in the central nervous system (CNS) [7]. The preference of using aluminum chloride (AlCl_3_) was compounded on the fact that it is found in many manufactured foods, toothpaste, medicines, and in purified drinking water [8]. Experimentally, long-term exposure to Al had been shown to cause not only neurological symptoms that mimic advanced neurodegeneration but also neurofilamental alterations in the cerebral cortex, hippocampus, brain stem, and spinal cord, in addition to biochemical changes observed in AD [9]. Thus, AlCl_3_-induced Alzheimer-like disease in rats is reported as the major commonly used animal model that mimics human AD [10].

The quest for natural therapeutic products that enhance cognitive performance and neuroprotection via antioxidant activation is of great interest, as many age-linked disturbances and neurodegenerative disorders are triggered by elevated oxidative stress [11]. One of the most promising natural products in this area is silymarin (SM), which is a medley of flavonolignans comprising silydianin, silybin, silychristin, and isosilybin and is extracted from the fruits and seeds of milk thistle (*Silybum marianum*) [12]. SM is regarded as the most effective drug for treating nearly all types of liver diseases, specifically alcoholic liver disease, chronic and acute viral hepatitis, and toxin-mediated liver impairments [13]. In addition to hepatoprotection, SM has been reported to prevent different kinds of cancers such as lung, bladder, prostate, breast, and ovarian cancers [14]. Recently, SM gained prominence as a neuroprotective compound as it has antioxidant and anti-inflammatory impacts in the CNS and can penetrate the CNS via the blood–brain barrier (BBB) [15]. Several studies reported that SM has neuroprotective influences in experimental models of ischemia, dementia, AD, and PD [16,17]. Furthermore, antidepressant and anxiolytic impacts of SM have been issued in rodents [18].

Therefore, the current investigation was designed to elucidate the neuroprotective impacts of SM on the hippocampal tissues of AlCl_3_-prompted Alzheimer-like disease in adult rats following biochemical, histological, and ultrastructural approaches.

## 2. Materials and Methods

### 2.1. Pharmacological Materials

Crystalline salts of AlCl_3_ and SM were bought from Sigma Aldrich Chemical Company (St. Louis, MO, USA). Moreover, other chemicals and reagents utilized in the current investigation were of analytical grade and highest purity.

### 2.2. Experimental Animals

Forty male Wistar rats (160–180 g) of similar age (12–16 weeks) were procured from the Research Institute of Theodor Bilharz, El-Giza, Egypt. These rats were kept in plastic cages provided with wood chips for bedding (2 animals/cage) under standard conditions (12 h light/dark cycle, temperature range of 25 ± 2 °C, and relative humidity of 55% ± 5%, besides water, milk, and food, which were provided ad libitum). Animals were habituated to these laboratory circumstances for seven days prior to the experiment. The followed protocol was confirmed by the local Institutional Animal Ethics Committee of Ain Shams University for the use and care of animals.

### 2.3. Experimental Protocol

The forty rats were categorized into four groups of 10 rats each.

Group I (Control group): some rats received vehicle for AlCl_3_, and others received vehicle for SM for 15 days.

Group II (SM group): on average, each rat was administrated daily 34 mg SM suspended in 1 mL corn oil (vehicle) for 15 days using oral gavage. This dosage adopted in the current study (200 mg/kg) also exhibited adequate protection in several sorts of brain disorders [16,19].

Group III (AlCl_3_ group): on average, each rat was intraperitoneally injected with 17 mg AlCl_3_ suspended in 1 mL distilled water (vehicle) once a day for 15 consecutive days. AlCl_3_ stock solution was prepared by dissolving the crystalline salts of AlCl_3_ in distilled water equivalent to 20 mg/mL and adjusted to pH 7.4 with 0.1 M phosphate buffer saline (PBS). This stock solution was used to prepare the applied dose of AlCl_3_, which was equivalent to 100 mg/kg. This dose was selected on the basis of studies published earlier [20,21].

Group IV (AlCl_3_ + SM group): rats were given 200 mg/kg/day SM concomitant with 100 mg/kg/day AlCl_3_ for 15 days in the same manner. 

### 2.4. Tissue Preparation

After the exploratory period, the animals were anesthetized with diethyl ether. The whole brains were dissected out on an ice plate and swilled with ice-cold isotonic physiological saline. Further, brains were divided into right and left hemispheres using a sharp blade for removing the hippocampi. The hippocampal tissues of 4 brains were randomly selected and processed for the histological and ultrastructural verification, while the weighed hippocampi of the rest of the brains were homogenized in ice-cold 0.1 mmol/L PBS (pH 7.4) using Teflon-glass homogenizer. The homogenates (10%, *w*/*v*) were then centrifuged at 10,000 rpm for 15 min and the supernatants were conserved at −80 °C for subsequent biochemical evaluations.

### 2.5. Biochemical Assessment

#### 2.5.1. Oxidative Stress Markers

Lipid peroxidation (LPO) in terms of formation of thiobarbituric acid reactive substances (TBARS) was colorimetrically measured at 532 nm following the previously described technique [22] and expressed as nmol malondialdehyde (MDA)/mg protein. 

Aggregation of nitrite, an indicator of the generation of nitric oxide (NO), in the supernatant was assessed using the colorimetric method as previously reported [23]. The obtained values were presented as µmol nitrite/mg protein.

Reduced glutathione (GSH) was measured following the previously described technique [24]. Absorbance of the developed yellow color was estimated at 412 nm against appropriate blank and the values were presented as nmol/mg protein.

The effectiveness of superoxide dismutase (SOD) was evaluated using an existing method [25] that is based on proportioning the SOD activity for inhibition of pyrogallol autoxidation at pH 8. The quantity of enzyme needed to produce 50% inhibition of the oxidation of pyrogallol corresponds 1 U of SOD activity. The obtained results were expressed as U/mg protein.

Catalase (CAT) activity was examined spectrophotometrically as previously depicted in reports [26], wherein the breakdown of H_2_O_2_ in the presence of CAT is estimated. Reduction in absorbance as a result of H_2_O_2_ degradations was monitored at 240 nm for 1 min, and CAT activity was expressed as nmol H_2_O_2_ consumed/min/mg protein.

#### 2.5.2. Assay of Acetylcholinesterase (AChE) Activity

AChE activity was estimated following the previously described protocol [27]. Under the effect of AChE, acetylthiocholine (ACh) is degraded into thiocholine and acetic acid. The catalytic activity is measured by monitoring the elevation of yellow anion, 5-thio-2-nitrobenzoate, that formed from the reaction of thiocholine with Ellman’s reagent at 410 nm. Results were presented as µmol of acetylcholine iodide hydrolyzed/min/mg protein.

#### 2.5.3. Proinflammatory Markers

Two proinflammatory cytokines, interleukin-1beta (IL-1β) and tumor necrosis factor-alpha (TNF-α), had been assessed in the supernatant using commercially accessible enzyme-linked immunosorbent assay (ELISA) kits subsequent to the instructions described by the manufacturer (eBIOSCIENCE, San Diego, CA, USA). The obtained values were presented in pg/mL.

#### 2.5.4. Protein Estimation

Protein content in each sample was assayed according to previously described procedures, using bovine serum albumin as a standard [28].

### 2.6. Histological Examination

The hippocampi of all rats were spliced into small segments and fixed for 24 h in Bouin’s fixative. Following the protocol previously described [29], all hippocampi samples were forwarded for paraffin sectioning. Subsequently, paraffin sections of 4–6 μm thick were stained with Ehrlich’s hematoxylin and eosin (Hx&E) stains, after that they were dehydrated using a sequence of graded concentrations of ethanol, cleared in xylene, mounted by DPX, examined, and photographed by a compound light microscope (BX-40 Olympus) attached with a camera (Panasonic CD-220).

### 2.7. Ultrastructural Examination

Tiny segments of the freshly excised hippocampi of all animal groups were immediately fixed for 24 h in cold 4F1G (4% formalin and 1% glutaraldehyde, pH 2.2), and post-fixed in 1% phosphate-buffered osmium tetroxide for 2–4 h. After that, they were managed for evaluation by the transmission electron microscopy (TEM) using the method originally defined by Dykstra et al. [30]. At the end of the procedures, the grids were examined and photographed by a JEOL.JEM-1200-EX-ELECTRON MICROSCOPE provided with a camera in the Central Laboratory of Faculty of Agriculture, Cairo University, El-Giza, Eygpt. 

### 2.8. Statistical Analysis

The biochemical results were shown as mean ± standard error of mean (SEM) of 6 samples per group. Statistical differences between groups were calculated by applying one-way analysis of variance (ANOVA) followed by Tukey post hoc test using the SPSS/20.0 software. A *p*-value < 0.05 was considered statistically significant.

## 3. Results

### 3.1. Biochemical Analysis

**Figure 1:**
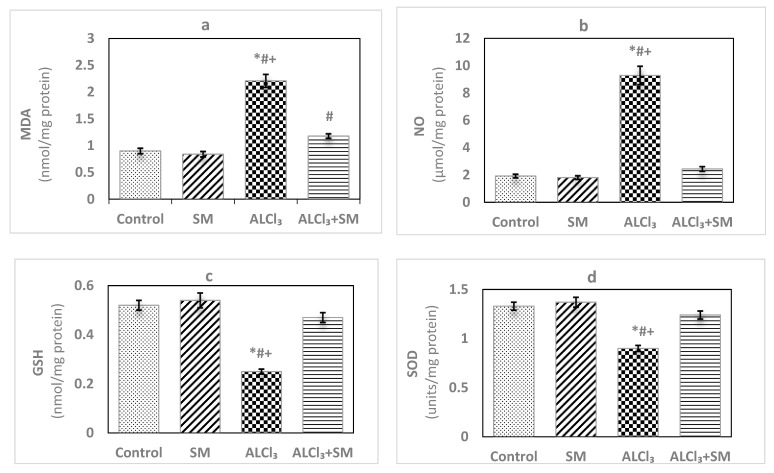

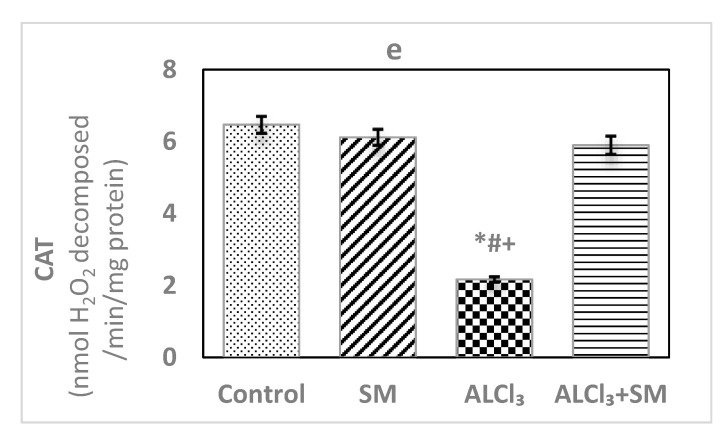
Levels of (**a**) malondialdehyde (MDA), (**b**) nitric oxide (NO) and (**c**) reduced glutathione (GSH), and activities of (**d**) superoxide dismutase (SOD) and (**e**) catalase (CAT) in hippocampal tissues of control and experimental groups of rats. Values are expressed as mean ± standard error of mean (SEM) (*n* = 6). Comparisons are as follows: * *p* ≤ 0.05, significantly different from Control; # *p* ≤ 0.05, significantly different from silymarin (SM); + *p* ≤ 0.05, AlCl_3_ significantly different from AlCl_3_ + SM group.

The effects of AlCl_3_ (100 mg/kg) intoxication and SM (200 mg/kg) treatment on LPO and oxidative stress indices of hippocampal tissues are represented in Figure 1. Administration of SM solely did not influence the values of these parameters when compared to the corresponding controls. Meanwhile, AlCl_3_-intoxicated animals showed marked increase (*p* ≤ 0.05) in MDA (145.56%) and NO (383.85%) levels along with significant decrease (*p* ≤ 0.05) in the level of GSH (−51.92%), besides activities of SOD (−32.33%) and CAT (−66.46%) in hippocampal tissues compared to control values. The toxic effects of AlCl_3_ on the measured indices were significantly (*p* ≤ 0.05) alleviated by administration of SM. 

**Figure 2:**
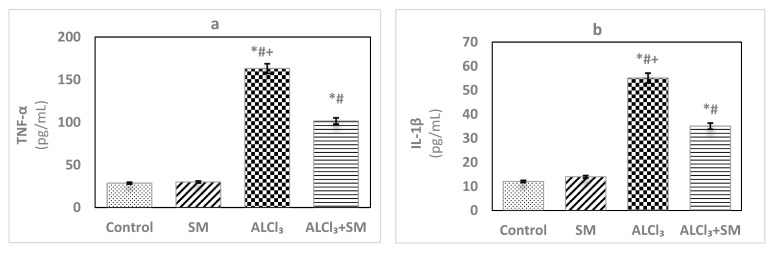
Levels of the proinflammatory cytokines (**a**) tumor necrosis factor-alpha (TNF-α) and (**b**) interleukin-1beta (IL-1β) in hippocampal tissues of control and experimental groups of rats. Values are expressed as mean ± SEM (*n* = 6). Comparisons are as follows: * *p* ≤ 0.05, significantly different from Control; # *p* ≤ 0.05, significantly different from SM; + *p* ≤ 0.05, AlCl_3_ significantly different from AlCl_3_ + SM group.

Under the current experimental conditions, administration of AlCl_3_ at a dose of 100 mg/kg (Figure 2) induced significant increase (*p* ≤ 0.05) of the proinflammatory cytokines TNF-α (469.63%) and IL-1β (356.93%) in hippocampal tissues compared to that in control rats. When a combined treatment with SM was applied in AlCl_3_-intoxicated animals, a significant decline (*p* ≤ 0.05) in the values of these markers was observed relative to the animals treated with AlCl_3_ alone, but these values were still significantly different (*p* ≤ 0.05) than the control ones. SM-treated rats did not show significant changes in the values of TNF-α and IL-1β as compared to control rats.

**Figure 3:**
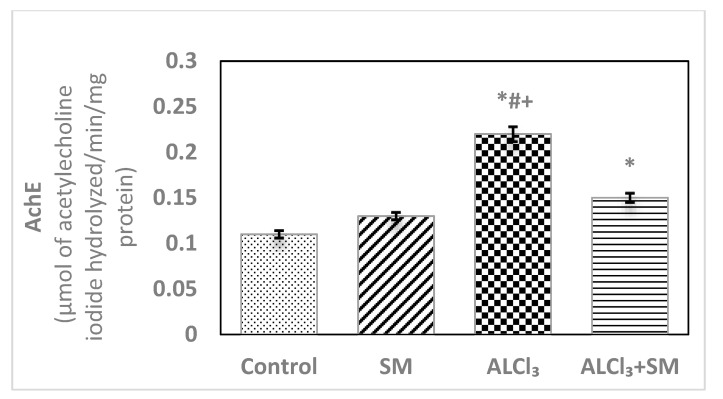
Levels of acetylcholinesterase (AChE) in hippocampal tissues of control and experimental groups of rats. Values are expressed as mean ± SEM (n = 6). Comparisons are as follows: * *p* ≤ 0.05, significantly different from Control; # *p* ≤ 0.05, significantly different from SM; + *p* ≤ 0.05, AlCl_3_ significantly different from AlCl_3_ + SM group.

Figure 3 depicts levels of AChE in hippocampal tissues of control and treated groups of rats. The obtained data exhibited changes in the values of this parameter in SM-treated group but in a non-significant manner when contrasted with the control group. Meanwhile, AlCl_3_ evoked significant rise (*p* ≤ 0.05) in AChE level (100%) in hippocampal tissues compared with that in the control animals. Supplementation of SM to the rats treated with AlCl_3_ caused modulation of this parameter compared to that in the animals subjected to AlCl_3_ alone; however, it was still significantly different (*p* ≤ 0.05) relative to the control values.

### 3.2. Histological Observations

**Figure 4:**
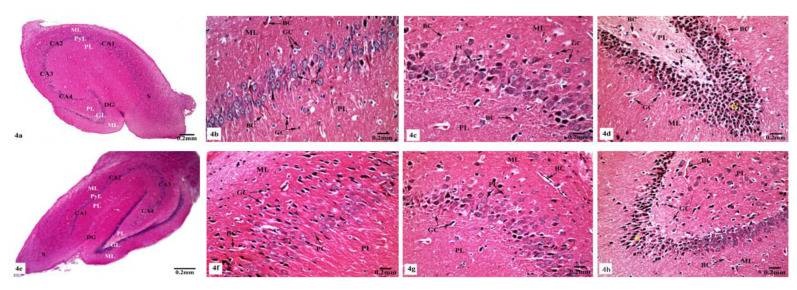
Photomicrographs of the hippocampal tissues of control (**a**–**d**) and SM-treated rats (**e**–**h**) stained with Hx&E showing (**a**,**e**) normal structure of cornu ammonis (CA), dentate gyrus (DG), and subiculum (S). CA is formed of the pyramidale layer (PyL) merged between the molecular layer (ML) and polymorphic layer (PL). CA1, CA2, CA3, and CA4 are subfields of the stratum pyramidale. The DG is seen surrounding CA4 by its upper and lower limbs and forming of molecular (ML), granular (GL), and polymorphic (PL) layers; (**b**,**f**) small pyramidal cells (PC) of the CA1 subfield appeared with large vesicular nuclei. Glial cells (GC) and blood capillaries (BC) are noticed in the molecular (ML) and polymorphic (PL) layers; (**c**,**g**) large pyramidal cells (PC) of CA3 region, most with vesicular nuclei. Additionally, glial cells (GC) and blood capillaries (BC) are seen; (**d**,**h**) compact closely packed granular cells (G) with dark stained nuclei are seen in the granular layer of the DG. Blood capillaries (BC) and glial cells (GC) are noticed in the molecular (ML) and polymorphic (PL) layers.

Light microscopic examination of Hx&E stained sections of the hippocampal tissues of rats from different groups showed consistent structure with easily identifiable cornu ammonis (CA), dentate gyrus (DG), and subiculum. As illustrated in Figure 4, the control (Figure 4a) and SM-treated rats (Figure 4e) revealed the C-shaped hippocampus, which is composed of the CA formed of stratum pyramidale sandwiched between stratum molecular externally and stratum polymorphic internally. The CA1, CA2, CA3, and CA4 subfields of stratum pyramidale were demonstrated. The DG is observed as a coiled structure with an opened concave part that was directed to the hippocampus proprius. The DG appeared to consist of three layers: an outer molecular layer with scattered small nervous cells, an intermediate granular layer with packed oval shaped nervous cells, and an inner polymorphic layer with multiform nervous cells. The CA1 and CA2 subfields of the hippocampal tissues in control (Figure 4b) and SM-treated rats (Figure 4f) showed small pyramidal cells that possess basophilic cytoplasm and large vesicular rounded nuclei with relatively pale stained dispersed nuclear chromatin materials and prominent nucleoli. Meanwhile, CA3 and CA4 zones of hippocampi of control (Figure 4c) and SM-treated rats (Figure 4g) were formed of large pyramidal cells with similarly large vesicular nuclei. Moreover, blood capillaries and small glial cells with small dark nuclei and undistinguished cytoplasm were seen in both stratum molecular and stratum polymorphic layers (Figure 4b,c,f,g). CA4 projects into the concavity of the DG, while the subiculum is an outward continuation of CA1 region (Figure 4a,e). The DG of the hippocampi of control and SM-treated rats appeared to consist of granular cells that were closely packed small cells with dark-stained nuclei. Blood capillaries and glial cells are illustrated in both the molecular and polymorphic layers of the DG (Figure 4d,h). 

**Figure 5:**
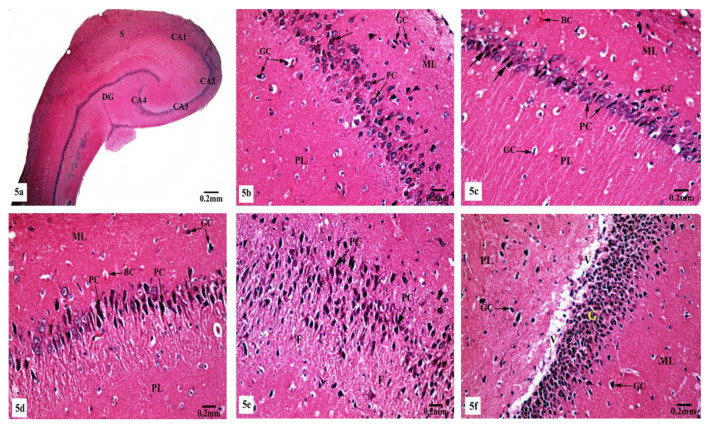
Photomicrographs of the hippocampal tissues of AlCl_3_-treated rats stained with Hx&E showing (**a**) disruption of normal laminar organization, where the hippocampus appeared flattened, formed of CA1, CA2, CA3, and CA4 subfields, DG, and subiculum (S); (**b**) the CA1 subfield had disorganized small pyramidal cells (PC) and some of them appeared shrunken with pyknotic nuclei (→). Some glial cells (GC) appeared enlarged in the molecular (ML) and polymorphic (PL) layers; (**c**) another CA1 zone revealed marked shrunken small pyramidal cells (PC) and some of them showed pyknosis (→). Dilated congested blood capillaries (BC) and glial cells (GC) were also seen; (**d**) the CA3 subfield revealed large pyramidal cells (PC) exhibiting shrinkage with pyknotic nuclei, moreover, enlarged glial cells (GC) and dilated blood capillaries (BC) were noticed; (**e**) disorganized large pyramidal cells (PC) having pyknotic nuclei, in addition to fibrosis (F) were clearly seen in the surrounding neuropil in another CA3 area; (**f**) the granular cell layer (G) of the DG showed marked vacuolation (V). Molecular (ML) and polymorphic (PL) layers showed marked enlarged glial cells (GC).

On the other hand, marked histological changes were observed in the hippocampal tissues of AlCl_3_-treated rats compared with those of the control and SM-treated rats as illustrated in Figure 5a–f. Figure 5a reveals the general structure of the hippocampus, which appeared flattened and formed of the CA, DG, and subiculum. CA1, CA2, CA3, and CA4 subfields of CA were seen. CA1 and CA2 subfields showed decreased thickness of the small pyramidal cell layer with disorganized small pyramidal cells, where some of them were shrunken with pyknotic nuclei, additionally, some glial cells of the molecular layer appeared enlarged (Figure 5b,c). Moreover, CA3 and CA4 subfields were severely affected as seen in Figure 5d,e, where large pyramidal cells appeared distorted with marked shrinkage in their sizes and having darkened nuclei. Fibrosis was clearly observed in the surrounding neuropil. Similarly, glial cells of the molecular layer were enlarged. The granular cell layer of DG showed marked vacuolation (Figure 5f). Furthermore, the molecular layer showed marked enlargement of glial cells (Figure 5f).

**Figure 6:**
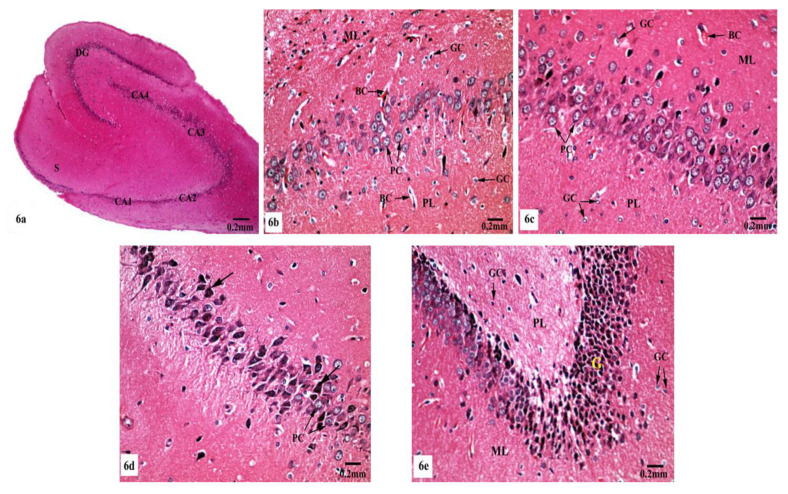
Photomicrographs of the hippocampal tissues of AlCl_3_ + SM-treated rats stained with Hx&E showing (**a**) restoration of the laminar organization with preservation of the characteristic structure of the hippocampal tissues: CA1, CA2, CA3, and CA4, DG, and subiculum (S); (**b**) preservation of small pyramidal cells (PC) of the CA1 subfield with normal glial cells (GC) and blood capillaries (BC) in the molecular (ML) and polymorphic (PL) layers; (**c**) the CA3 subfield appeared with nearly normal large pyramidal cells (PC) having vesicular nuclei, additionally intact glial cells (GC) and blood capillaries (BC) were seen; (**d**) another CA3 area revealed large pyramidal cells (→) having dark nuclei among normal ones (PC); (**e**) preservation of normal organization of granular cells (G) of the DG with normal glial cells (GC) in both polymorphic (PL) and molecular (ML) layers.

Meanwhile, supplementation of SM to the rats treated with AlCl_3_ caused restoration of the laminar organization with the preservation of the characteristic structure of the hippocampal tissues (Figure 6a–e). The CA1, CA2, CA3, and CA4 subfields, DG, and subiculum of the hippocampi appeared nearly normal (Figure 6a). Figure 6b reveals the preservation of the small pyramidal cell architecture of CA1 and CA2 subfields, which appeared with normal glial cells and blood capillaries in the molecular and polymorphic layers. CA3 and CA4 subfields appeared with nearly normal-sized large pyramidal cells with normal organization and vesicular nuclei, and normal glial cells and blood capillaries are seen in Figure 6c. Furthermore, another CA3 area revealed large pyramidal cells having dark nuclei among normal ones (Figure 6d). No diffused vacuolar degeneration was observed in the granular layer of the DG of the hippocampal tissues. Additionally, molecular and polymorphic layers showed normal-sized glial cells and widened blood capillaries (Figure 6e).

### 3.3. Ultrastructural Observations

**Figure 7:**
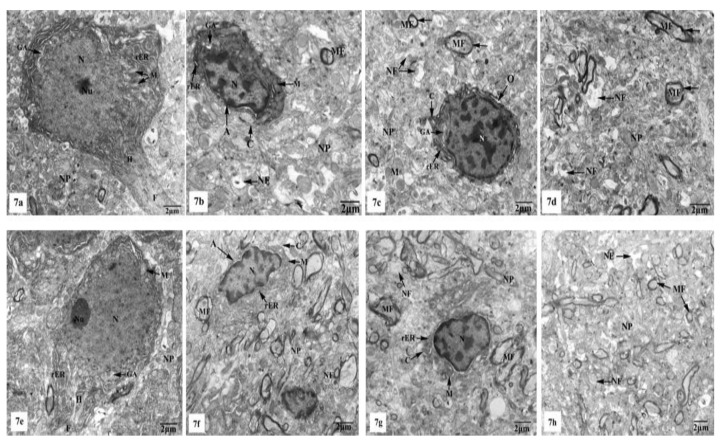
Electron micrographs of the hippocampal CA1 subfields of control (**a**–**d**) and SM-treated rats (**e**–**h**) showing (**a**,**e**) pyramidal neurons possessing huge irregular nuclei (N) with dispersed chromatin and prominent nucleoli (Nu). Their cytoplasm appeared rich in rough endoplasmic reticulum (rER), mitochondria (M) with normal densities, and Golgi apparatus (GA). Moreover, the axon hillock (H) and its initial part with normal distribution of neurofilaments (F) and intact surrounding neuropil (NP) are clearly noticed; (**b**,**f**) astrocytes (A) containing irregular electron-dense nuclei and cytoplasm having few rER, Golgi apparatus (GA), and mitochondria (M), in addition to cytoplasmic extensions (C) can be identified. Furthermore, adjacent neuropil (NP) containing normal myelinated (MF) and non-myelinated (NF) fibers is observed; (**c**,**g**) oligodendrocytes (O) having electron-dense ovoid nuclei (N) and cytoplasm with rER and Golgi apparatus (GA), in addition to cytoplasmic extensions (C) are seen. The surrounding neuropil (NP) comprising some myelinated (MF) and non-myelinated (NF) axons is also observed; (**d**,**h**) numerous myelinated (MF) nerve fibers with regular myelin sheaths (←) or non-myelinated (NF) nerve fibers are seen in the neuropil (NP).

Electron micrographs of the hippocampal CA1 region of the control (Figure 7a–d) and SM-treated rats (Figure 7e–h) showed pyramidal neurons with the ordinary fine structures. Each neuron possesses a cell body that has a large irregular and centrally located nucleus containing dispersed chromatin and prominent nucleolus, and its cytoplasm appears rich in rough endoplasmic reticulum (rER), mitochondria, and Golgi apparatus. Moreover, the neuron axon with its hillock and normal allocation of neurofilaments is clearly seen (Figure 7a,e). Two principal types of neuroglia cells are clearly recognized: astrocytes and oligodendrocytes. Electron micrographs (Figure 7b,f) revealed normal astrocytes, which possess irregular, elongated, and electron-dense nuclei and thecytoplasm comprise few cisternae of rER, Golgi apparatus, and mitochondria. Furthermore, the provenance of several cytoplasmic extensions can be recognized. Dark oligodendrocytes are also illustrated in Figure 7c,g, they have rER, mitochondria, and Golgi stacks in the cytoplasm, in addition to electron-dark rounded nuclei. The commencement of cytoplasmic processes of these oligodendrocytes can be observed. The surrounding neuropil includes several neuronal and glial processes in different sectional planes. Multiple nerve fibers either myelinated, which are surrounded by consistent myelin sheaths with conserved orderly thickened lamellar structure, or non-myelinated nerve fibers, with numerous mitochondria, neurofilaments, and microtubules are obviously seen (Figure 7d,h). 

**Figure 8:**
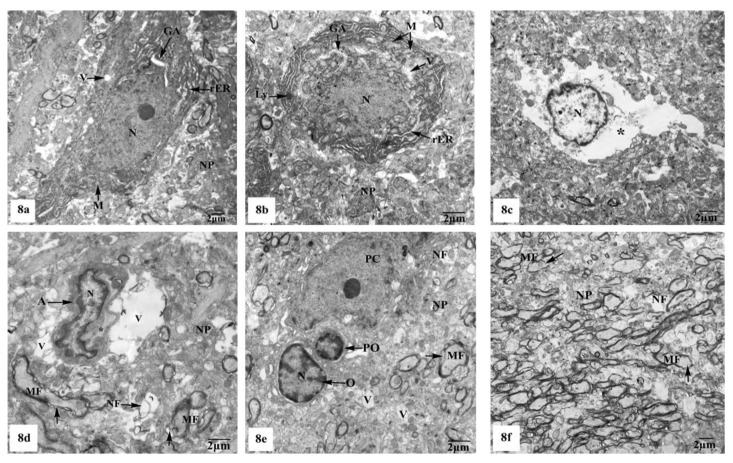
Electron micrographs of the hippocampal CA1 subfields of AlCl_3_-treated rats showing (**a**) a pyramidal nerve cell with irregular-shaped dispersed nucleus (N) and deformed cytoplasm illustrating dilated rER, swollen Golgi apparatus (GA), electron-dense mitochondria (M), and cytoplasmic vacuoles (V); (**b**) another distorted neuron with irregular shrunken nucleus (N), swollen cisternae of rER, swollen mitochondria (M) with their cristae partly missing, dilated Golgi apparatus (GA), lysosomes (Ly), and cytoplasmic vacuolation (V); (**c**) a severe degenerated neuron appeared with small heterochromatic nucleus (N) and vacuolated cytoplasm (*) with cytoplasmic remnants; (**d**) a deteriorated astrocyte (A) appeared with highly wrinkled nucleus (N) and cytoplasmic vacuolation (V). The surrounding neuropil (NP) revealed vacuolation (V), and myelinated nerve fibers (MF) with discontinuity of their myelin coats (←) are also seen; (**e**) two oligodendrocytes lying adjacent to the pyramidal nerve cell (PC) with one of them appearing pyknotic (PO) and the other one (O) possessing a small electron-dense nucleus (N). Furthermore, myelinated nerve fibers (MF) with discontinuity of their myelin coats (←) are seen in the neuropil (NP); (**f**) aggregated myelinated nerve fibers (MF) having abnormal or irregular thickened and arranged myelin sheaths (←), in addition to irregular non-myelinated nerve axons (NF) are clearly seen in the neuropil (NP).

On the other hand, the hippocampal CA1 subfields of the rats treated with AlCl_3_ showed marked alterations compared to those of control and SM-treated rats as clearly observed in Figure 8a–f. As illustrated in the electron micrographs (Figure 8a,b), pyramidal neurons appeared with irregular dispersed nuclei and altered cytoplasmic organelles represented by dilated rER, swollen Golgi apparatus, electron-dense mitochondria, and lysosomes, in addition to cytoplasmic vacuoles. Severe destruction was seen in another neuron, where its nucleus appeared shrunken with an irregular contour and the cytoplasm revealed reduction of the intracellular organelles including mitochondria, rER, and Golgi apparatus, only cytoplasmic vacuoles and cytoplasmic remnants are seen as clearly observed in Figure 8c. Neuroglia cells especially astrocytes and oligodendrocytes also revealed degenerative changes of similar intensity to those in nerve cells. Deteriorated astrocytes with highly wrinkled nuclei and cytoplasmic vacuolation are illustrated in Figure 8d. Oligodendrocytes revealed signs of pyknosis which were reflected by shrunken electron-dense nuclei and destructed cytoplasmic components are distinguished in Figure 8e. In addition, severe degenerative alterations affecting mainly the myelinated nerve fibers were visible, where some of them showed abnormal thickening and irregular arrangement of their myelin coats and the others revealed focal lysis of their myelin sheaths. Additionally, irregular non-myelinated nerve axons are clearly seen in the neuropil (Figure 8f).

**Figure 9:**
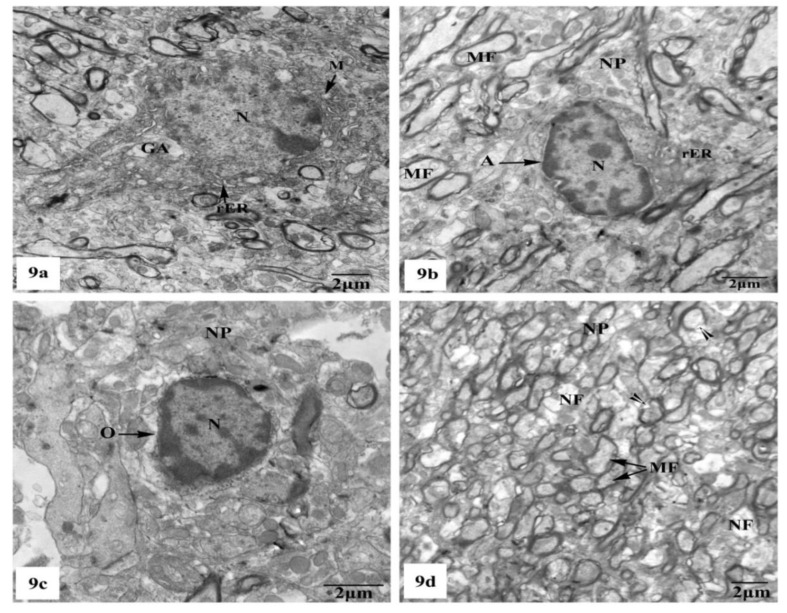
Electron micrographs of the hippocampal CA1 subfields of AlCl_3_ + SM-treated rats showing (**a**) nearly normal pyramidal nerve cell which possesses irregular nucleus (N) with dispersed chromatin and the cytoplasm containing less electron-dense mitochondria (M), rER, and Golgi apparatus (GA); (**b**) an astrocyte (A) with nearly normal electron-dense nucleus (N) and cytoplasm having few cristae of rER. The neuropil (NP) with myelinated nerve fibers (MF) is seen; (**c**) an oligodendrocyte (O) with nearly normal cytoplasm and nucleus (N); (**d**) a part of the neuropil (NP) with nearly normal myelinated (MF) and non-myelinated (NF) nerve fibers. Other myelinated nerve fibers appeared with discontinuous and split abnormal myelin sheaths (arrow heads).

Whereas TEM examination of the hippocampal CA1 area of the rats treated with AlCl_3_ concomitant with SM exhibited partial improvement of most of the fine structural characteristics of the neurons and neuroglia cells as clearly seen in Figure 9a–d; some myelinated nerve fibers still showed discontinuity of their myelin sheaths from their axons (Figure 9d). 

**Figure 10:**
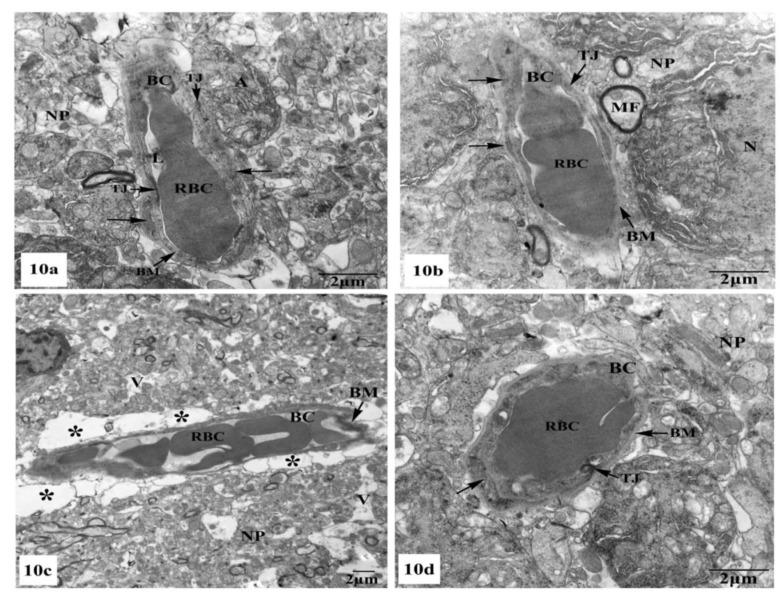
Electron micrographs of the blood–brain barrier (BBB) in the hippocampal CA1 subfields showing (**a**,**b**) intact blood capillaries (BC) with narrowed lumens (L) containing red blood cells (RBCs) and appearing with continuous and integrated basement membranes (BM) that have normal tight junctions (TJ) and surrounded by many astrocyte processes (←) in control and SM-treated rats, respectively; (**c**) a blood capillary (BC) surrounded with greatly expanded astrocyte processes (*) and its basement membrane was disrupted and the tight junctions were unclear, besides, the surrounding neuropil (NP) revealed vacuolation (V) are seen in AlCl_3_-treated rats; (**d**) nearly normal blood capillary (BC) enclosing red blood cells (RBCs) in its narrowed lumen and surrounded with intact basement membrane (BM) showing tight junctions (TJ), in addition, the surrounding foot processes (←) of the adjacent astrocyte appeared normal in AlCl_3_ + SM-treated rats.

Further ultrastructural examination of the BBB in the hippocampal CA1 region of all animal groups was performed. In control (Figure 10a) and SM-treated rats (Figure 10b), the BBB revealed normal blood capillaries with narrowed lumens and vascular endothelial cells that showed normal cell structures with continuous and integrated basement membranes and tight junctions. The extensions of astrocytes revealed no swelling. However, rats treated with AlCl_3_ exhibited noticed ultrastructural alterations in the BBB of their hippocampal CA1 region as seen in Figure 10c. The blood capillaries appeared enlarged with increased lumens, and their surrounding basement membranes were loosened, and their densities became uneven. Additionally, the tight junctions were unclear. The foot processes of astrocytes appeared swollen and the surrounding neuron fibers also swelled (Figure 10c). On the other hand, treatment with SM paralleled with AlCl_3_ in rats revealed nearly normal BBB with recovered blood capillaries having intact basal laminas with tight junctions that were identified as normal. The foot processes of astrocytes appeared nearly normal (Figure 10d).

## 4. Discussion

Al is reported as a potent neurotoxin and has been associated with AD since it exacerbates brain oxidative injury, causes neuronal inflammation, and induces Aβ deposition, which leads to impairment in working memory [9,31]. It accelerates LPO and induces increased free radical generation, thereby causing oxidative stress, which results in severe neurotoxicity [32]. The brain is known to be most susceptible to the adverse influences of Al, and it is especially vulnerable to oxidative stress resulted from elevated levels of free radicals and diminished levels of antioxidants following toxicity [33]. A large group of neurodegenerative diseases, characterized by progressive loss of central neurological function, has been recorded in infancy and childhood [34]. Therefore, the present research was performed to study the potential neuroprotective role of SM against AlCl_3_-induced Alzheimer-like disease in early adult rats through biochemical, histological, and ultrastructural analyses. 

Free radicals are a group of highly reactive molecules, which form as natural derivatives of biochemical reactions occurring inside the living cells. These substances comprise reactive nitrogen (RNS) and oxygen (ROS) species and they can react readily with the cellular macromolecules such as proteins, carbohydrates, lipids, and nucleic acids causing huge harm to cell structures [35]. Living cells produce endogenous anti-oxidative factors, which buffer the generated free radicals and provide protection to the cells against oxidative injury. The most prominent endogenous antioxidant molecules are GSH, SOD, and CAT [36]. When the free radicals produced exceed the ability of the cell to neutralize them through antioxidant molecules, a condition called oxidative stress occurs [37]. Levels of MDA and NO are other important indicators of oxidative stress in biological systems. MDA is produced by peroxidation of membrane lipids caused by ROS, which causes damage and degradation of the membrane [38]. NO serves as a neurotransmitter molecule in the CNS, but when it is generated in excess, it will be neurotoxic [39].

In the current study, administration of AlCl_3_ resulted in marked oxidative stress in the hippocampal tissues as indicated by rising the levels of LPO and NO and decreasing the levels of GSH, retarding the activities of CAT and SOD. Induction of oxidative stress in brain tissues in response to sub-chronic exposure to Al has been confirmed in previous works accomplished by many authors [9,40]. The nuclear factor-erythroid-2-related factor 2 (Nrf2) is a vital factor contributed in the organization of redox homeostasis by raising the expression of various antioxidant-related genes, including those of SOD, CAT, and GSH [41]. Previous studies confirmed that dysregulation of Nrf2 signaling is associated with AlCl_3_-induced hippocampal lesions in rats [42]. In the present study, SM alone did not have any effect on the oxidative stress markers in hippocampi of normal rats; however, it significantly attenuated AlCl_3_-induced oxidative damage. SM is known to possess a strong antioxidant activity, and it can restore the redox balance due to its free radical scavenging ability, thus preventing peroxidation of membrane lipids [43]. In the same context to our results, the neuroprotective influence of SM against oxidative stress in the brain of rat has been manifested by previous studies [44]. The strong antioxidant potential of SM may be attributed to its ability to activate Nrf2 signaling and induce the expression of antioxidant enzymes [45]. 

The inflammation cascade has a vital role in the pathogenesis of neurodegenerative diseases, including AD [46]. IL-1β and TNF-α are proinflammatory cytokines that contribute to immune dysfunction and mediate inflammation of the tissues and organ injury [47]. In the existing study, intoxication of rats with AlCl_3_ markedly elevated TNF-α and IL-1β in the hippocampal tissues, suggesting that AlCl_3_ preferentially affects macrophage functions and stimulates the development of brain injury. Our results are compatible with the previous studies reporting neuroinflammation following exposure to Al [48,49]. Nuclear factor-kappa B (NF-κB) is a protein complex that plays a pivotal role in the activation of the neuroinflammatory cascade causing transcription of IL-1β and TNF-α [50]. Compelling evidence has supported that AlCl_3_-induced neuroinflammation may be an outcome of acute oxidative stress due to ROS-mediated NF-κB activation [51]. The present study revealed significant suppression of the over-production of IL-1β and TNF-α in hippocampal tissues of AlCl_3_-intoxicated rats treated with SM. The anti-inflammatory potential of SM has long been recognized as it causes inhibition of a variety of inflammation-related signaling pathways including the NF-κB pathway [52].

ACh is a cholinergic neurotransmitter that has a key role in the transmission of neural signals between neurons and associated with the construction and maintenance of learning memory in the brain. AChE is the enzyme involved in hydrolyzing ACh to choline and acetate [53]. The present study manifested that AlCl_3_ caused a significant elevation in the activity of AChE. This finding is in accordance with previous investigations [20,54]. The onset of AD begins with the lack of ACh and, therefore, suppressing the activity of AChE, which elevates ACh level, has positive impact on the cognitive function [55]. Here, co-administration of SM to AlCl_3_-intoxicated rats exhibited neuroprotection by reducing the activity of AChE. In the same context, Kiruthiga et al. [56] and Nazir et al. [57] previously confirmed that SM can increase the content of ACh through inhibiting the activity of AChE, thereby alleviating the AD condition.

Our biochemical findings were confirmed by the histological and ultrastructural studies, where AlCl_3_ administration in rats induced severe hippocampal damage, particularly in the CA and DG regions. The current findings showed pronounced degeneration of pyramidal neurons and neuroglia cells, in particular astrocytes and oligodendrocytes. Marked degenerative changes also affected the myelinated nerve fibers, where some of them exhibited irregular configuration of their myelin coats, while the others revealed focal degranulation of their myelin sheaths. The presence of dark electron-dense neurons in the current results represents a certain apoptosis, which is characterized by strikingly intense nucleoplasm and cytoplasm. This result coincided with previously reported finding [58]. On the other hand, Sobaniec-Lotowska [59] assumed that the dark small neurons are typically ischemic, resulting from severe defects in capillary walls with consequent disorders in the structure of the BBB. However, Carageorgiou et al. [60] reported that these degenerated neurons tended to result from disruption of several proteins, enzymes, nucleic acids, and multiple neurotransmitter biosynthesis. Mitochondrial and nuclear defects were considered secondary to the overt toxicity of the neuronal cells causing biochemical abnormalities [61]. Dilatation of the rER may happen due to lipid peroxidation [62]. 

Cytoplasmic vacuolation in the distorted nerve cells of AlCl_3_-treated rats may be due to lipid peroxidation, in addition to damage of the cell membranes and membranes of some organelles. This damage is accompanied by an elevation in sodium permeability that raises the capacity of the pump to extrude sodium. The aggregation of sodium in the cell results in elevated water content in the cytoplasm, causing its swelling [63]. Furthermore, neuropil vacuolation may be attributed to neuronal cell shrinkage, in addition to the degeneration of their processes leaving pericellular spaces. These suggest neuronal necrosis as manifested in early stages of excitotoxic, ischemic, hypoxic/ischemic, and hypoglycemic conditions [64]. Meanwhile, the neuropil vacuoles could be resulted from swollen neuronal processes and presynaptic nerve endings, whereas the cytoplasmic vacuoles were related to swollen mitochondria [65].

Moreover, the swollen astrocyte processes could be caused as a result of lipid peroxidation and increasing in sodium permeability, which lead to sodium accumulation inside the cells and are sequenced by an elevation in water content and swelling of these cells [63,66]. The astrocytes perform vital roles in neurotransmitter metabolism and uptake, neurotransmitter receptor expression, neurotrophic-factor secretion, extracellular matrix protein secretion, myelination, and synaptic integration [67].

The anomalies in myelin sheaths found in this study could be explained as a consequence of oxidative stress that is popular characteristic of axonal atrophy, in addition to differences in myelin thickness, which can affect the conservations of the circular shapes of these myelinated fibers [68]. Furthermore, the free radicals could disrupt the oligodendrocytes, which are responsible for myelination, leading to damage of their cell membranes and impairment of myelination. Such degenerative alterations in the nerve cells axons were in consent with Gerspacher et al. [69], who recorded deterioration of the cytoskeleton in cadmium (Cd)-treated rats, and with Rai et al. [70], who reported similar hazardous influences induced by Cd in the myelinated nerve fibers of cerebral cortex, retina, and optic nerves. The changes in the axons were identified as part of the dying process of neuronal injury, while the disruption of the myelination was related to alterations in the myelin base protein secondary to axonal deterioration and degeneration. 

The BBB is a permeablebarrier located between the brain tissue and peripheral blood. It is a diffusion membrane formed of a complex network of brain capillary endothelial cells and pericytes connected with each other by a basement membrane that surrounding the astrocytes end feet [71]. This compact multiple overlapping layer prohibits toxic substances in the blood from reaching the brain tissue, controlling the blood–brain niche internal and external environments, stabilizing the water balance and preserving the normal functions of the CNS [72]. The extensions of astrocytes form the second barrier of BBB that is composed of a layer of tough glial membrane covering 99% of the brain capillaries’ surfaces. Both barriers are important for the preservation of BBB function. Astrocytes have been reported to perform vital roles in the formation of vascular endothelium and in the maintenance of the integrity of vascular structure [73]. BBB disruption may be related to β-amyloid clearance and proinflammatory cytokine transport. This disturbance could be linked to the progression of various CNS disorders, such as AD and cognitive impairment [74]. 

In the current study, after AlCl_3_ therapy, ultrastructural damage in the BBB was demonstrated, as the endothelial cells and astrocytic extensions were significantly altered. Parallel with our results, Flaten [75] and Maria Rob et al. [76] demonstrated that the primary lesion in AD, dementia, and PD resulted from impaired BBB permeability, which allowed Al to enter the CNS. Al can accumulate in the brain with Al–citrate and Al–transferrin complexes entering the BBB [77]. At the same time, the elevated levels of Al contribute to an extensive increase in BBB permeability by altering its fine structure [78,79]. Furthermore, degeneration of the neurons resulted in changes in the BBB permeability that causing leakage of albumin, which acts as a shield guarding the brain against several harmful agents [80]. 

Given our histological and ultrastructural results, SM may be useful in reducing hippocampal tissue injury in AlCl_3_-intoxicated rats. Since, SM caused attenuation of most of the alterations induced by AlCl_3_. The majority of neurons, their axons, and myelin sheaths and even neuroglia cells were approximately similar to those in the control group. Moreover, treatment with SM improved the BBB ultrastructure of rats intoxicated with AlCl_3_. This was probably related to SM anti-oxidative, anti-inflammatory, and cytoprotective properties [43]. The ability of SM to cross the BBB supports its usage as a neuroprotective agent [15,81], and ameliorates glutathione in the brain resulting from oxidative stress and aged-linked and pathological degenerative processes [15,82]. 

Concomitant with the current results, SM was also issued to be neuroprotective versus oxidative insults in the hippocampi and cortices of the elderly rodents by potentially suppressing the formation of peroxyl and oxygen radicals together with protein oxidation products [19]. SM was found to relieve the cognitive deterioration triggered by Aβ-accumulation through prohibiting the oxidative damage in the hippocampi of mouse brains, regarding lipid peroxidation and glutathione levels and also attenuating the over-expression of inflammatory mediators such as TNF-α [83]. Additionally, the protective effect of SM on Aβ-accumulation was designated to the blockade of its aggregation, which indicates that SM strongly inhibits Aβ-dependent neuropathology via its anti-amyloidogenic ability [84]. Moreover, SM recompensed manganese- and acetaminophen-mediated oxidative stress and neurotoxicity in animal models by increasing the activities of enzymatic and non-enzymatic antioxidant markers [85]. Furthermore, SM delayed neuronal cell death in the hippocampal tissues of rats after ischemic surgery [86]. Mehri et al. [87] emphasized the potent antioxidant properties of SM and its neuroprotective effects against acrylamide-induced toxicity in both in vivo and in vitro animal models. Moreover, there is convincing evidence that most of the Al in serum is bound to the iron-transport protein transferrin and a very small amount binds to albumin [88]. It has also been shown that SM stimulates protein synthesis in liver cells [89]. Therefore, overproduction of serum proteins in response to SM treatment, which then bind to the excess Al in serum, cannot be ruled out. Moreover, monocarboxylate transporters are membrane carriers involved in the efflux of Al from brain regions [77]. An increase in expression of these carriers within the brains of mice has been confirmed by Pierre et al. [90]. Therefore, corn oil given to group IV could also be of a beneficial role in the structural improvement seen in the brains of rats of this group.

## 5. Conclusions

The present experimental study suggests that SM may be useful in reducing hippocampal tissue injury of AlCl_3_-kindled rats due to its antioxidant and anti-inflammatory properties.
